# Characterization of the Viral Reservoirs Among HIV-1 Non-B Vertically Infected Adolescents Receiving Antiretroviral Therapy: Protocol for an Observational and Comparative Study in Cameroon

**DOI:** 10.2196/41473

**Published:** 2022-11-30

**Authors:** Aubin Joseph Nanfack, Georgia Elna Ambada Ndzengue, Joseph Fokam, Aude Christelle Ka'e, Nelson Sonela, Leslie Kenou, Michelle Tsoptio, Bertrand Sagnia, Elise Elong, Grace Beloumou, Carlo Federico Perno, Vittorio Colizzi, Alexis Ndjolo

**Affiliations:** 1 International Reference Center Chantal Biya Yaounde Cameroon; 2 Department of Animal Biology and Physiology University of Yaounde Yaounde Cameroon; 3 Faculty of Health Science University of Buea Buea Cameroon; 4 Faculty of Medicine and Biomedical Sciences University of Yaounde Yaounde Cameroon; 5 Department of Immunology University of Rome Tor Vergata Rome Italy; 6 Medical School University of KwaZulu-Natal Durban South Africa; 7 Department of Microbiology and Parasitology Protestant University of Central Africa Yaounde Cameroon; 8 Faculty of Medicine and Biomedical Science University of Dschang Dschang Cameroon; 9 Bambino Gesu Children's Hospital Rome Italy

**Keywords:** HIV, viral reservoirs, adolescents, vertical infection, Cameroon

## Abstract

**Background:**

Antiretroviral therapy (ART) can bring HIV-1 levels in blood plasma to the undetectable level and allow a near-normal life expectancy for HIV-infected individuals. Unfortunately, ART is not curative and must be taken for life, because within a few weeks of treatment cessation, HIV viremia rebounds in most patients except for rare elite or posttreatment controllers of viremia. The primary source of this rebound is the highly stable reservoir of latent yet replication-competent HIV-1 proviruses integrated into the genomic DNA of the resting memory cluster of differentiation 4 (CD4+) T cells. To achieve a cure for HIV, understanding the cell reservoir environment is of paramount importance. The size and nature of the viral reservoir might vary according to the timing of therapy, therapeutic response, ART duration, and immune response. The mechanisms of reservoir maintenance generally depend on the levels/type of immune recognition; in addition, the dynamics of viral persistence are different between pediatric and adult populations. This difference could become more evident as children grow toward adolescence.

**Objective:**

We aim to characterize the HIV reservoirs and their variability as per the virological and immunological profiles of HIV-1 non-B vertically infected adolescents receiving ART in Cameroon during the Adolescents' Viral Reservoirs study to provide accurate and reliable data for HIV cure research.

**Methods:**

This study will involve HIV-1 non-B vertically infected adolescents selected from an existing cohort in our institution. Blood samples will be collected for analyzing immunological/virological profiles, including CD4/CD8 count, plasma viral load, immune activation/inflammatory markers, genotyping, and quantification of HIV-1 viral reservoirs. We will equally recruit an age-matched group of HIV-negative adolescents as control for immunological profiling.

**Results:**

This study received funding in November 2021 and was approved by the national institutional review board in December 2021. Sample collection will start in November 2022, and the study will last for 18 months. The HIV-1 sequences generated will provide information on the circulating HIV-1 subtypes to guide the selection of the most appropriate ART for the participants. The levels of immune biomarkers will help determine the immune profile and help identify factors driving persistent immune activation/inflammation in HIV-infected adolescents compared to those in HIV-uninfected adolescents. Analysis of the virological and immunological parameters in addition to the HIV-1 reservoir size will shed light on the characteristics of the viral reservoir in adolescents with HIV-1 non-B infection.

**Conclusions:**

Our findings will help in advancing the knowledge on HIV reservoirs, in terms of size and genetic variability in adolescents living with HIV. Such evidence will also help in understanding the effects of ART timing and duration on the size of the reservoirs among adolescents living with HIV—a unique population from whom the findings generated will largely contribute to designing functional cure strategies.

**International Registered Report Identifier (IRRID):**

PRR1-10.2196/41473

## Introduction

### Importance and Relevance

Sub-Saharan Africa is disproportionally affected with HIV/AIDS, with close to 70% of the global epidemic and the highest burden of pediatric HIV infections; about 9 out of every 10 children living with HIV is found in the Sub-Saharan African region [1,2]. Due to combination antiretroviral therapy (ART) benefits, adolescents and young people represent a growing share of people living with HIV worldwide. In 2019, about 1.7 million adolescents between the ages of 10 and 19 years were living with HIV worldwide, representing about 5% of all people living with HIV; about 1.5 million or 88% of HIV-infected adolescents live in Sub-Saharan Africa. In 2019 alone, 460,000 young people between the ages of 10 and 24 years were newly infected with HIV, of whom 170,000 were adolescents. Of the estimated 690,000 people who died of AIDS-related illnesses in 2019, 110,000 (or approximately 16%) of them were children younger than 20 years, including 32,000 aged 10-19 years [2,3].

With the advent and scalability of ART, there is a global decrease in AIDS-related deaths. As of June 2021, 28.2 million [3] people were accessing ART, representing 73% of all people living with HIV. However, only 54% of the children living with HIV were receiving ART. Interestingly, about 94% of all the children receiving ART are from Sub-Saharan Africa [4]. In this context of continuous new HIV pediatric infections and increasing coverage in pediatric ART, the number of children living with HIV will increase, suggesting a higher likelihood of reaching adolescent age and even adulthood if treatment regimens remain fully effective in controlling HIV infections [3,4]. Adolescents living with HIV (ADLHIV) therefore constitute an HIV population with growing health concerns and with very limited findings for generalizable best practices specific to this target population, especially in Sub-Saharan Africa.

Cameroon still faces a generalized HIV epidemiology (2.7% prevalence) [5], with higher prevalence among pregnant women (5.7%) and HIV-exposed infants/children (5.8% positivity at first polymerase chain reaction [PCR] and 15% at the end of the prevention of mother-to-child HIV transmission cascade) [6]. As of December 2020, the national coverage of ART was 74% (or 367,871 HIV positive), which includes 12,017 children (35% coverage) younger than 15 years [6]. Regarding response to ART in Cameroon, an overall rate of 79.4% viral suppression was reported, with significant disparities across age ranges: 81.1% in adults, 75.6% in children, and only 53.3% in adolescents aged 10-19 years [7]. Similar to that reported by the Joint United Nations Programme on HIV/AIDS, ADLHIV represent the most vulnerable and underserved population in response to the epidemic [1,5]. For a safer growth toward adulthood, there is need to prioritize this population for the quest of innovative treatment strategies that ensure their well-being and their contribution to the development of Sub-Saharan Africa.

Despite the unquestioned benefits of ART, there are limitations with current treatment strategies. Of note, the lifelong nature of current ART goes with challenge-related adherence for most patients, ART-attributed toxicities and persisting immune dysfunction in patients lead to significant health impairments, and HIV drug resistance is increasing, mostly in Sub-Saharan African countries where most ART-experienced patients are living [8]. There is a threat of an emerging new HIV epidemic, driven by HIV drug resistance to existing antiretrovirals. These challenges are particularly true for pediatric populations due to limited ART options, poor drug formulations, and increasing events of nonadherence as they grow toward adolescence. These challenges call for approaches toward HIV (functional) cure or remission, especially for the most vulnerable populations (ie, ADLHIV) [7,9].

### Concepts Underpinning the Project Including Ideas and Models or Assumptions

HIV-1 remission or eradication strategies aim to achieve durable control of the virus in the absence of ART. The development of an HIV-1 cure remains challenging due to latent reservoirs. The latter can be defined as the fraction of cells harboring transcriptionally silent proviral DNA that can produce infectious virions following activation [10]. Resting memory cluster of differentiation 4 (CD4) T cells are the primary host of the latent reservoir, but HIV-1 infection in these cells is inefficient due their low coreceptor expression and inherent restrictions to reverse transcription [11,12]. The provirus is maintained in a latent state in these cells via host factors due to integration into the expressed genes [13]. Viral rebound from the latent reservoir following ART cessation is rapid, leading to detectable viremia within weeks of therapy interruption [14]. Initiating ART early during infection is not sufficient to stop the formation of the latent reservoir, suggesting that the latent reservoir is established and disseminated early [15], even in vertically infected children who started ART soon after birth [16]. Despite years of suppressive ART, the latent reservoir is stable and is the source of rebound viremia following therapy interruption. Latently infected cells therefore represent a critical barrier to HIV-1 cure. The progress toward the development of a functional or sterilizing cure (virus remission/eradication) for HIV-1 has been significantly hindered by the presence of the latent reservoir. Therefore, understanding of where and how HIV persists in individuals on ART has transformed substantially with evidence that the virus persists in multiple cell types and tissue sites. Thus, in the frame of virological success during ART, accurate estimates of the viral reservoir would help in better mastering of viral persistence, which in turn might overcome existing barriers for achieving a complete cure [17].

Total HIV DNA is a reference biomarker that includes both integrated and unintegrated HIV DNA and reflects the global level of the viral reservoir. Buzon et al [18] reported a statistical correlation between the time from the start of the HIV infection to treatment initiation and the total HIV DNA level after 10 years of continuous treatment in a cohort of adults first treated with early infection [19]. In children, the HIV DNA level was markedly lower when viral control was achieved before the age of 1 year [18]. By comparison with other markers, total HIV DNA has the advantage of easy quantification by standardized, sensitive, real-time PCR, including digital droplet PCR [20].

Generalized immune activation is typically related to HIV-1 infection. A variety of immune cells show an increase in the expression of activation and production of proinflammatory cytokines [21,22]. Immune activation is associated with HIV-1 disease progression; suppression of viral replication with effective ART reduces immune activation, but even effective ART regimens are unable to bring it to levels seen in healthy individuals [21]. HIV-infected children, even if successfully treated with ART regimens, face a lifetime of elevated immune activation; evaluating the potential impact of this chronic immune activation and inflammation on their immune system and on disease outcome is very important [23]. Recent studies showed that immune activation and exhaustion markers are strongly associated with the reservoir size in ART-treated adults; thus, it might be anticipated that minimizing the viral reservoir with early ART might equally minimize the level of immune activation [23,24].

There is limited evidence in characterizing HIV reservoirs in the western and central African region—a geographical setting having the highest variability in circulating HIV-1 and HIV-2 strains [25,26]. For example, Cameroon, a zoonotic epicenter of HIV-1, is host to an extensively diverse landscape of HIV driven by the CRF02_AG recombinant, including most group M (sub-) subtypes, a vast array of unique recombinant forms, circulating recombinant forms, group N, group O, group P, and HIV-2 viruses [27-29]. Thus, generating baseline data on the genotypic and quantitative profile of the viral reservoir across several HIV clades in settings like Cameroon would inform the design of optimal strategies for HIV cure. Considering the aforementioned vulnerability of adolescents with vertical infection and the limited knowledge on viral reservoirs and immune activation/inflammatory reaction in this population, evidence generated from this target will be highly complementary to current global efforts. Such evidence, generated in a context of high burden of coinfections [30,31], might depict differential mechanisms of HIV persistence far from those reported in other parts of the world. 

### Preliminary Work

Within the frame of the European and Developing Countries Clinical Trial Partnership-Resistance Evolution among Adolescents in Yaoundé and its surroundings (EDCTP-READY) study (Multimedia Appendix 1), we have set up a cohort of 292 vertically infected adolescents (10-19 years) receiving ART in Cameroon. In this cohort, we reported a rate of 40% undetectable viral load (<40 copies/mL) after a median of 8 years of ART, about 20% immunological failure (CD4<250 cells/mm^3^) rate, and less than 10% clinical failure (ie, World Health Organization stages III/IV) [32]. This population offers a unique opportunity for understanding the size and nature of the reservoir; the variability of immune response/cytokine profiling; and the effect of the viral subtype, treatment history of ART (regimen and duration), gender disparities, and adherence level on the control of the viral reservoir. To date, most HIV cure research has been restricted to high-income countries with relatively low HIV burden and has most often engaged men who have sex with men. HIV strains are genetically and biologically diverse, and host mechanisms of antiviral immunity required for durable control may differ by age, sex, geography, and ethnicity. Basic discovery research and clinical trials in resource-limited settings must be strengthened to contribute to the global cure strategy [33]. Our study aims to characterize the HIV reservoirs and their variability according to the virological and immunological profiles of vertically infected adolescents receiving ART in Cameroon and therefore improve the understanding of the viral reservoirs and provide accurate and reliable data for HIV cure research in settings harboring broad genetic diversity. In this study, we shall (1) determine HIV-1 genetic variability and drug resistance in cellular reservoirs, (2) determine the immune profile of ADLHIV, (3) quantify their HIV viral reservoir, and (4) evaluate the effect of ART and immune response on the viral reservoir profile.

## Methods

### Study Design

We plan to conduct a cross-sectional, observational, and comparative study among vertically infected ADLHIV receiving ART in Cameroon. Participants will be selected and enrolled from an existing cohort of close to 300 vertically infected adolescents recruited for the EDCTP-READY study [7]. They will provide written assent and legal guardians will provide written proxy consent. HIV positive adolescents with incomplete ART history and hepatitis B virus/hepatitis C virus and malaria coinfections will not be considered for this study.

### Sample Size

The minimum sample size was estimated at 90 participants, assuming an HIV prevalence of 2% in adolescents in Cameroon, a 95% confidence, and 80% statistical power. The sample size was further stratified into 3 arms of 30 participants each: arm A, HIV viral load<40 copies/mL; arm B, HIV viral load=40-999 copies/mL; and arm C, HIV viral load≥1000 copies/mL. A group of 30 HIV-negative adolescents will serve as control for immunological profiling.

### Study Procedures and Variables

#### Procedures and Timelines

This study requires 18 months to be completed: 3 months (month 1-3) for administrative and ethics approvals; 12 months (month 4 to 15) for enrolment of participants, sampling, and laboratory analyses; and 3 months (month 16 to 18) for data curing, processing, and reporting.

#### Sampling Strategy

Based on inclusion criteria, HIV positive adolescents case report forms will be selected from the EDCTP-READY study and their legal guardians will be contacted and invited to the clinic. Study clinicians will obtain new assent and informed consent from adolescents and legal guardians, respectively. Sociodemographic data, clinical data, and complete ART history will be collected, and eligible participants will be enrolled. Intravenous blood (5 mL × 2) will be collected by a trained phlebotomist for analyses in the central laboratory (Chantal Biya International Reference Center for Research on Prevention and Management of HIV/AIDS [34]).

#### Laboratory Procedures

Samples will be collected only once at enrolment along with all the relevant sociodemographic and clinical data. Samples will be transported to the central laboratory on the same day within 6 hours to be processed. Samples will be used for CD4/CD8 measurements (absolute counts and percentages), immune activation/inflammatory markers assessment by flow cytometry (FACSCanto II, BD BioSciences), and plasmatic viral load by real-time PCR (Abbott m2000rt). Peripheral blood mononuclear cells will be isolated by density-gradient centrifugation. HIV DNA will be subsequently extracted from peripheral blood mononuclear cells by using the QIAamp DNA Mini Kit (Qiagen), stored at –80 ^o^C, and shipped every 3 months to the Department of Experimental Medicine, University of Rome Tor Vergata for HIV viral reservoir quantification using a homemade droplet digital PCR [35]. Proviral DNA will be extracted from the buffy coat by using the DNeasy blood and tissue extraction kit (Qiagen), and HIV viral RNA will be extracted from participants with virological failure (viral load>1000 copies/mL) using the QIAamp viral RNA Mini Kit (Qiagen); HIV proviral DNA and HIV viral RNA will be subsequently genotyped following a homemade protocol [36] on a 3500 genetic analyzer (Applied Biosystems). DNA sequences will be analyzed for drug resistance mutations by using the Stanford University database genotypic resistance interpretation algorithm [37]. For phylogenetic analysis, neighbor joining phylogenetic trees will be created using the Molecular Evolutionary Genetics Analysis software (Kimura 2-parameter model, 200 bootstrap replications) and FigTree [38,39].

#### Data Collection and Analysis

Data analysis will be performed under the responsibility of the project’s biostatistician and the supervision of the principal investigator. Data will include sociodemographic data, clinical data, and laboratory data. Standardized case report forms will be generated for onsite data collection and laboratory results. Case report forms will be completed by the authorized clinic and laboratory personnel under the supervision of the principal investigator and the co–principal investigators. All data will be entered onsite through a double entry system in a password-protected computer, and case report forms will be kept in a locked office only accessible to authorized project personnel. Each participant will be assigned a unique identifier. Data will be analyzed using SPSS software (IBM Corp). Associated factors will be evaluated using multivariate logistic regression, with an estimate approach for the unbiased effect of different parameters. The data will be reported as medians. Nonparametric tests will be used for data not normally distributed. Comparisons of medians among different groups (virological success vs virological failure) will be performed using the Mann-Whitney *U* test. Correlations will be made with Spearman test, and *P* values less than .05 will be considered statistically significant.

### Ethics Approval and Consent to Participate

This study will be conducted per the declaration of Helsinki on ethical principles for medical research involving human subjects. A written proxy-informed assent from legal guardians and a written assent from the participating HIV positive adolescent will be obtained without any coercion. Privacy and confidentiality will be ensured through the use of unique identifiers and a password-protected database accessible only by authorized staff. Participants will be free to deliberately leave the study at any time, without any effect on their routine monitoring at the study clinic. Phlebotomy will be noninvasive (venipuncture) and will be performed by a trained nurse. Ethical clearance has been obtained from the Cameroon National Ethics committee for research on human health (No2021/12/1426/CE/CNERSH/SP).

### Management of Potential Risks

The risks to the participants are minimal since the only procedure the volunteer is subjected to, is venipuncture by a phlebotomist or physician. The venipuncture may be slightly painful, but it is practically without any risk of complication. The potential risks to subjects, none of which are likely to occur, may include momentary pain and bruising at the site or possible (but extremely unlikely) infection. If such complications arise, participants will be provided with emergency medical care.

### Quality Assurance

Our study team will include a quality assurance officer who will be responsible for all the standard operating procedures for the study protocol and who will manage proficiency testing and data validation during the entire study. Quality assurance will be assessed by external proficiency testing for plasmatic viral load, CD4/CD8 count, and genotyping.

## Discussion

Despite the current success of ART (66% viral control globally) [3], lifelong treatment is required because there is no cure. On the basis that not everyone can access and adhere indefinitely to ART, a global consensus emerged several years ago that a curative intervention was a high priority to bring an end to the HIV pandemic [33]. Within a few weeks of treatment cessation in individuals with undetectable plasmatic viral load, HIV viremia rebounds in most patients, except for rare elite or posttreatment controllers of viremia; the primary source of this rebound is the highly stable reservoir of latent yet replication-competent HIV-1 proviruses integrated into the genomic DNA of resting memory CD4+ T cells [17,40]. To achieve a cure for HIV, understanding the cell reservoir environment is one of the key topics to address as prerequisite for the development of successful cure strategies and interventions [33].

The first specific objective of this study, that is, to determine HIV-1 genetic variability and drug resistance in cellular reservoirs, will help inform if the development of HIV drug resistance mutations in circulating viruses correlates with mutations found in latent reservoirs for possible eradication considerations and therefore, guides the selection of the most active ART with potential impact on latent reservoirs.

The second specific objective, that is, to determine the immune profile (activation/inflammation status) of the study participants, will help identify factors driving persistent immune activation, particularly during immunological/virological success/failure in vertically infected adolescents. Additional research is needed to conclusively state whether there are clear differences in the effects of specific ART history or HIV-1 subtypes on inflammation and immune activation in ADLHIV.

The third and fourth specific objectives, which are to quantify HIV reservoirs and evaluate the effect of ART and immune response on the reservoir profile, will inform if the characteristics of the latent reservoirs are associated with immune activation/inflammation, HIV-1 subtypes, and treatment outcome (virological success vs virological failure), especially in adolescents and therefore call for specific management of this sensitive population.

Overall, our findings will help in advancing knowledge on the HIV reservoir, in terms of size, genetic variability, and immune profile in ADLHIV. Such evidence will also help in understanding the effects of ART timing and duration on the size of reservoirs among ADLHIV—a unique and vulnerable population from whom the findings generated will largely contribute to designing functional cure strategies.

## Appendix

Multimedia Appendix 1 Peer review report by European & Developing Countries Clinical Trials Partnership (EDCTP2) - Horizon 2020 (European Union).

## Abbreviations

ADLHIV: adolescents living with HIV
ART: antiretroviral therapy
CD: cluster of differentiation
EDCTP-READY: European and Developing Countries Clinical Trial Partnership-Resistance Evolution among Adolescents in Yaoundé and its surroundings
PCR: polymerase chain reaction

## Data Availability

The data sets used and analyzed during this study will be available from the corresponding author on reasonable request. HIV gene sequences generated during this study will be publicly available in the GenBank database [42].
