# Supplementary material for: Characterization of the Viral Reservoirs Among HIV-1 Non-B Vertically Infected Adolescents Receiving Antiretroviral Therapy: Protocol for an Observational and Comparative Study in Cameroon
Source: JMIR Res Protoc. 2022 Nov 30;11(11):e41473. doi: 10.2196/41473 (PMC9752448; doi:10.2196/41473)
Supplement: Multimedia Appendix 1 [file resprot_v11i11e41473_app1.pdf]

European & Developing Countries Clinical Trials Partnership

**Reference**	**TMA2020CDF-3228**
**Title**	**Characterisation of Viral Reservoirs among HIV-1 non-B Vertically Infected Adolescents receiving Antiretroviral Therapy in Cameroon**
**Lead Applicant**	**Dr Aubin Nanfack**
**Organisation**	**International Reference Centre Chantal Biya (CIRCB)**
**Reviewer**	**5**

Assessment - Rapporteur

Review

Excellence

Excellence - Review Comments

- **Fit with the scope and objectives of the EDCTP2 programme, the EDCTP Association strategic research agenda and the call topic description.**

The project fits within the scope of the EDCTP2 programme. The project is important and the objectives are clear.

- **Importance, relevance/pertinence and clarity of the objectives.**

As HIV research pushes towards a cure, understanding the size and nature of the HIV reservoir in those taking ART is of fundamental importance; and conducting that research across all clades of HIV will be necessary. This research gains credit from being conducted in Cameroonian adolescents who were vertically infected with non-clade B virus; and from being able to compare the characteristics of the reservoirs across those with viral suppression and those with replicating virus.

- **Soundness of the concept and credibility of the proposed approach/methodology.**

The concept is sound, lab-based and with credible methodology. The study is important and relevant as it has the potential to provide knowledge of viral reservoirs. This study can also provide accurate and reliable data that may lead to the reduction or elimination of the HIV reservoir, thereby contributing to HIV cure research as well as research into new therapeutics or interventions for HIV-infected individuals.

A weakness is that the key technique for measuring reservoir size will be conducted at the mentor's university

in Rome, with no plan to transfer the technology to the candidate’s laboratory. A further weakness is that only a single reservoir measurement was proposed, perhaps include the novel IPDA assay that scores the biologically relevant active reservoir. Although the project itself is strong, the panel felt that the investigator would be better off employing a statistician to undertake the analysis, rather than learning statistics himself. The statistician would then ensure that the investigator understands the basis of the statistical analysis, rather than aiming to do this themselves.

There is no clinical trials template.

There were a few methodological weaknesses:

1. The number and HIV subtypes (non-B subtypes, O, N, and P viruses and HIV-2) expected is missing.
2. The sample size needs clarity as the reviewers felt that n=90 was a very general number.
3. Clarity is needed to understand if the proposed virus assays are able to pick up these non-B viruses or whether they have to be adjusted.
4. Clarity is needed on how the controls will be recruited.
5. The candidate has highlighted lack of funding as an issue, but has only applied for 18 months of funding. Further funding could have led to more interesting and indepth investigations.

- **Suitability of the candidate, considering their track record, degree of independence and/or potential, and how the fellowship will further the individual’s career.**

The fellow has a PhD in immunology and microbiology (2015) and heads up the laboratory in his institution in Cameroon. His CV lists several international positions (Rome, Cote d'Ivoire, Germany, New York, New York) and he has worked as local principal investigator on a study in collaboration with Columbia University as part of his post-doctoral diploma, but has not led a grant of his own. There is a certain lack of detail about why previous placements were so short and how they built the candidate’s skills. This suggests that there may have been a lack of opportunity to develop within previous posts. The fellow has 3 first author papers and four posters.

The fellow has not clearly articulated a vision for the future as a scientist. The many activities have provided technical expertise in several directions but training in scientific thinking and hypothesis development is lacking. This should be developed if this fellowship is awarded.

- **Quality of the project and its fit with the fellow’s expertise and career development plan, including acquired competencies and skills to be developed further.**

The candidate plans training in biostatistics, grant writing and is looking forward to leading his first research project, including completing the protocol, ethics submissions and implementation of the work. He intends to employ and train two lab technicians and two Masters students during the fellowship (and retain them as PhD candidates in his own research group thereafter). The panel felt that the candidate should employ a statistician rather than planning on undertaking the statistics himself.

- **Quality of the mentorship and/or training plan.**

The mentor is established in the field of HIV research, and has participated in the new droplet PCR technology developed for quantifying the HIV reservoir and is therefore appropriate to guide the candidate in his chosen field of study. The mentorship is distance mentoring. This may well be appropriate for the stage that the investigator has reached but particularly with data and grant writing a closer relationship might be beneficial. The panel felt that the mentoring should be clearly described and more frequent than quarterly.

**Excellence - Score**	3.5

Impact

Impact - Review comments

- **Call specific aspects as listed under 'expected impact' in each individual call.**

The impact of the study could have international implications and would advance the field.

- **The extent to which the outputs of the proposed work would contribute, at the European, African and/or International level, to each of the expected impacts listed in the work plan under the relevant topic.**

The project is novel, and although focused on basic science, the resultant improved understanding of the viral reservoir and immune responses in those successfully vs non-successfully treated with ART has great potential. In the absence of an HIV vaccine, research strategies in HIV cure have gained traction. Eradication or cure of HIV requires in-depth knowledge about HIV latent reservoirs and the focus on local African non-B clade subtypes is most important locally. The project will provide information on HIV reservoirs that are particularly important in developing strategies to eliminate or eradicate HIV. Information from this project has the potential to provide information on additional therapeutics to reduce inflammation and systemic immune activation in virally suppressed HIV-infected individuals. However, the impact may only be on increasing research knowledge and may not directly benefit the affected populations. Further advances could be gained if the laboratory techniques and skills needed to build and support HIV cure research in Africa could be transferred to the local team.

- **Likelihood to result in major advances in the field with the potential benefit of the research to the affected populations.**

Overall, the panel felt that as the fellow is building on past work with his current mentor, is already a skilled laboratory technician, and is using an already existing cohort, it is likely that the candidate will achieve the study goals even within the short 18-month time frame proposed.

- **Advancing the fellow's clinical research skills and career development.**

Career development is sub-optimal to date and the courses described are quite general. Some more specific

courses provided by his mentors might be beneficial to demonstrate that the candidate has the potential to move to research independence. The panel would like clarity on the names and roles of the Co-PIs described in the application.

It would be useful to clarify what happens to the head of diagnostics post that the applicant holds whilst they undertake the fellowship or would it be expected that this continues concurrently, in which case, how will the investigator have time to undertake the study?

- **Contribution to development of research independence and/or scientific leadership.**

The fellowship will increase research independence if the mentoring is strengthened.

- **Contribution to strengthening clinical research capacity at the home or host organisation.**

The letters of support are favourable. It appears that the training of the candidate will bring new skills to the institution, for example, there is currently no biostatistician available. Biostats training has been identified as a key area for the candidate; thus generating a skill that will enhance local research capacity. However, it may be that rather than training in yet another new skill, the fellow recruits a statistician who can then train others. There is undoubtedly a need for capacity in Cameroon for immunologists and the proposal is based in country which is a particularly strong point. The proposal could be strengthened if the proposed assays could be transferred to Cameroon. There does not appear to be any plans for increasing clinical research capacity.

- **Effectiveness of the proposed measures to exploit and disseminate results generated during the fellowship (including management of IPR), to communicate the fellowship activities, and, where relevant, to manage clinical data.**

There are plans for dissemination to scientific and national communities through meetings and presentations as well as through publications. The communication and dissemination sections could be strengthened by including the adolescent participants and their parent stakeholders in the feedback sessions.

- **Sustainability and retention of capacity beyond the end of the grant.**

The 2-year contract extension after the fellowship was not mentioned, probably because the candidate already has a permanent position.

**Impact - Score**	3.5

Quality and Efficiency of Implementation

**Implementation - Review comments**

- **Quality and effectiveness of the work plan, including the extent to which the resources assigned to work packages are in line with their objectives and deliverables;**

The proposed workplan is concise, clear and in line with the objectives and deliverables. Some clarification is required to understand whether the workplan is effective:

1. Ethics - what is the consent process in Cameroon and what Youth Services are available to sensitise the adolescents to the study and help around stigma? The clinical trial template was not completed and so a full review of ethical implications is required.
2. The quality control of data checks could be more detailed.
3. It is not clear how the investigator will split their time between being the head of the immunology lab and this fellowship. This could pose a risk to the project, especially as the investigator is currently single-handed.
4. The candidate and his team will require GCP training.

The staff budget is modest. There are appropriate resources set aside for specimen transport and analysis; and for analysis and publication.

- **Appropriateness of the management structures and procedures, including risk and innovation management, and how responsibilities for research data quality and sharing, and security will be met.**

The management structures and procedures are well-defined and appropriate, although clarity of the 2 Co-PIs is required. The fellow will plan and coordinate the study on a daily basis (75% effort) with support from another investigator (40%) who will focus on the clinical aspect of recruitment and sample collection, which is appropriate considering the lab-based expertise of the candidate. As already mentioned, it is not clear what will happen to the fellow's role as laboratory head during the fellowship and whether this could impact on study delivery. The data management and quality structure is well described and there is budget for a quality assurance officer to ensure the work produced is of high quality.

- **Complementarity of the participants within the consortium, and the extent to which the consortium as whole brings together the necessary expertise.**

There is support planned for the current coordinators of the EDCTP-READY cohort from which this study's cohort will be drawn.

- **Appropriateness of the allocation of tasks and resources, ensuring that all participants have a valid role and adequate resources in the project to fulfil that role.**

The home institution has the operational capacity to carry out the proposed work. The mentors have the competence and experience to guide the candidate to effectively complete this project.

- **Feasibility and appropriateness of the methods and project management to achieve the objectives within the timeframe of the grant.**

Feasibility is good as many routine assays are proposed.

- **Compliance with national and international standards of research, Good Clinical Practice, ethics and safety related issues.**

The study complies with standards of research but requires an ethics assessment and consideration of consent/assent and sensitisation and stigma within the adolescent groups that has not been considered.

- **Participants have the operational capacity, to carry out the proposed work, based on the competence and experience of the individual participant(s).**

The participants have the operational capacity to carry out the work, care needs to be taken about the current head of laboratory position the fellow holds.

- **Suitability of the fellow's home and/or host organisation to support the fellowship project.**

Both his mentor and host institution are in support of this candidate and project. The host institute has the assays required for the study. The candidate has worked with his mentor in the past. There are plans for regular calls for mentorship purposes, but a stronger mentoring program should be made available to the candidate to try to move him from a routine laboratory technician to an early career researcher.

- **Intention of the fellow's home organisation to develop and commit to a career post-fellowship or reintegration plan.**

The candidate is permanent staff at the home organization and will likely continue on as an independent researcher after this fellowship at his home institution. The panel anticipates that the fellow now needs to move to independence.

**Implementation - Score**	3.5

**Total Score**	10.5
